# Intranasal Immunization of Baculovirus Displayed Hemagglutinin Confers Complete Protection against Mouse Adapted Highly Pathogenic H7N7 Reassortant Influenza Virus

**DOI:** 10.1371/journal.pone.0063856

**Published:** 2013-06-07

**Authors:** Subaschandrabose Rajesh Kumar, Syed Musthaq Syed Khader, Tanja K. Kiener, Milene Szyporta, Jimmy Kwang

**Affiliations:** 1 Animal Health Biotechnology, Temasek Lifesciences Laboratory, National University of Singapore, Singapore, Singapore; 2 Department of Microbiology, Faculty of Medicine, National University of Singapore, Singapore, Singapore; Leiden University Medical Center, The Netherlands

## Abstract

**Background:**

Avian influenza A H7N7 virus poses a pandemic threat to human health because of its ability for direct transmission from domestic poultry to humans and from human to human. The wide zoonotic potential of H7N7 combined with an antiviral immunity inhibition similar to pandemic 1918 H1N1 and 2009 H1N1 influenza viruses is disconcerting and increases the risk of a putative H7N7 pandemic in the future, underlining the urgent need for vaccine development against this virus.

**Methodology/Principal Findings:**

In this study, we developed a recombinant vaccine by expressing the H7N7-HA protein on the surface of baculovirus (Bac-HA). The protective efficacy of the live Bac-HA vaccine construct was evaluated in a mouse model by challenging mice immunized intranasally (i.n.) or subcutaneously (s.c.) with high pathogenic mouse adapted H7N7 reassorted strain. Although s.c. injection of live Bac-HA induced higher specific IgG than i.n. immunization, the later resulted in an elevated neutralization titer. Interestingly, 100% protection from the lethal viral challenge was only observed for the mice immunized intranasally with live Bac-HA, whereas no protection was achieved in any other s.c. or i.n. immunized mice groups. In addition, we also observed higher mucosal IgA as well as increased IFN-γ and IL-4 responses in the splenocytes of the surviving mice coupled with a reduced viral titer and diminished histopathological signs in the lungs.

**Conclusion:**

Our results indicated that protection from high pathogenic H7N7 (NL/219/03) virus requires both mucosal and systemic immune responses in mice. The balance between Th1 and Th2 cytokines is also required for the protection against the H7N7 pathogen. Intranasal administration of live Bac-HA induced all these immune responses and protected the mice from lethal viral challenge. Therefore, live Bac-HA is an effective vaccine candidate against H7N7 viral infections.

## Introduction

H7N7 is a subtype of influenza virus A, a genus of *Orthomyxoviridae* and one of the causative agents of fowl plague in poultry. Subtype H7 influenza viruses fall into 2 distinct genetic lineages based on geography, e.g. North American or Eurasian viruses, both of which are associated with multiple outbreaks in poultry and human infections since 2002. H7 subtype viruses exist as both high and low pathogenic strains based on their ability to cause disease in chicken. H7N7 can infect humans, birds, pigs, seals, and horses in the wild, and has infected mice in laboratory studies. This unusual zoonotic potential represents a pandemic threat for humans in the future. Before 2002, H7 human infection cases were very rare and most could be traced back to occupational accidents or laboratory exposures [1] while there have been more than 100 cases documented since. The largest outbreak of subtype H7 (NL/219/03) human infection occurred in February 2003 in the Netherlands where 89 cases were detected, following an outbreak in poultry on several farms [2]. A further 3 family members who had not been in contact with infected poultry also contracted the virus which suggests that human to human transmission of the avian virus had occurred [2]. Most of these individuals suffered from conjunctivitis or mild influenza like illness, but one fatal case (FC) was recorded as a result of pneumonia and acute respiratory syndrome [2].

The attachment pattern of influenza FC H7N7 (NL/219/03) virus to the human respiratory tract showed great similarity to H5N1 virus [3]. The H7N7 virus can infect mice and ferrets without adaptation and has a lysine in position 627 of the basic polymerase 2 gene [3] which allows it to efficiently replicate in mammalian cells like H5N1 viruses and to cause severe disease and death following human infection. A previous report found that H7N7 viruses from both North American and Eurasian lineages exhibited a delayed and diminished induction of innate immune responses during infection in human lung cell lines [4]. The broad host spectrum of high pathogenic (HP) H7N7 coupled with its ability to suppress host immune responses in a similar fashion to 1918 H1N1 [5] makes vaccine development against this virus a priority to improve preparedness for a possible pandemic.

Previous vaccine studies against this virus reported that i.n. immunization of live attenuated vaccine strain [6] and recombinant low pathogenicity (LP) vaccine strain with adjuvant [7] protected the mice adequately. However, the live attenuated vaccine has the possible risks of reversion and recombination with circulating seasonal flu. Also, the low pathogenicity vaccine strain with adjuvant may cause adverse reactions if the vaccine formulation reaches the central nervous system through the olfactory nerves. Here we developed a live recombinant subunit vaccine using the HA protein of H7N7 (NL/219/03) virus together with baculovirus as a vector system for HA protein expression and as a delivery vehicle for HA into mammalian cells.

Influenza hemagglutinin (HA) is the major surface protein for virus attachment and fusion and has been extensively used for influenza virus subunit vaccine production. The recombinant HA vaccine approach is an attractive alternative method for vaccine production because it removes the need for egg- or cell-based replication of live influenza virus thus eliminating the associated requirement for biosafety levels 2+ or 3 for facilities and equipments. Several previous studies reported that insect cell or plant cell derived recombinant HA protein vaccine protects chicken, mice and ferrets against the H5N1 virus [8], [9], [10]. Here baculovirus was used as a vector system for HA protein expression in Sf9 insect cells and to deliver the expressed protein into mammalian cells.

The baculovirus *Autographa californica* nucleopolyhedrosis virus (AcNPV) is a double-stranded DNA virus that naturally infects insects but not mammals [11]. In recent years, AcNPV has been widely used as a novel vector for gene therapy and vaccine development [12]. The baculovirus surface displayed system has several attractive characteristics: (i) efficient production of complex recombinant proteins that require folding and other posttranslational modifications like glycosylation, (ii) transduction of mammalian cells without the ability to replicate in them [13], (iii) strong adjuvant properties through activation of dendritic cell (DC) mediated innate immunity through the MyD88/Toll like receptor 9 pathway [14]. Furthermore, baculovirus can stimulate IFN production from both human and mouse cells and confer protection from lethal encephalomyocarditis virus infections in mice [15]. The strong innate immune response produced by baculovirus also protects mice from a lethal challenge of influenza A and B viruses [16]. Baculovirus has been used as a vaccine vector for several animal and human diseases and was shown to induce both humoral and cell mediated immunity [10], [17], [18], [19].

The aim of the present study was to develop a potential recombinant vaccine against highly pathogenic influenza virus H7N7 (NL/219/03) by expressing the HA protein of H7N7 virus on the surface of baculovirus (Bac-HA). The vaccine efficacy was evaluated by induction of immune responses and protection in BALB/c mice immunized by subcutaneous (s.c.) or intranasal (i.n.) route followed by intranasal challenge with mouse adapted HP H7N7 reassortant virus.

## Materials and Methods

### Ethics statement

All animal experiments were carried out in accordance with the Guidelines for Animal Experiments of the National Institute of Infectious Diseases (NIID). Experimental protocols were reviewed and approved by Institutional Animal Care and Use Committee of the Temasek Life Sciences Laboratory, National University of Singapore, Singapore. (IACUC approval number TLL-12-001). Mice were housed in individually ventilated cages (Tecniplast Sealsafe) provisioned with water and standard food, and monitored daily for health and condition. More than 25% body weight loss was used as criterion for early euthanasia. The animals were euthanized by CO_2_ inhalation for five minutes.

### Viruses and cell lines

Reassortant influenza virus H7N7 (NL/219/03) was generated by reverse genetics as described previously [20]. For the synthesis of reassortant virus, the complementary DNA of the HA and NA genes of the H7 virus (NL/219/03) were synthesized based on the sequences from the NCBI influenza database while the six cDNAs of the internal genes were synthesized based on the PR8 (A/Puerto Rico/8/1934) virus sequence (GenScript, USA). The cDNA of each of the eight influenza virus gene segments was inserted between the pol I promoter (pIh) and the pol I terminator of pClpolsaplT vector (kindly provided by Ruben Donis, CDC, USA) and cotransfected into cocultured 293T and MDCK cells using lipofectamine 2000 (Life tech, USA). Madin–Darby canine kidney (MDCK) cells were maintained in Dulbeccos Modified Eagle Medium (DMEM; Life Technologies, USA) containing 10% Fetal Bovine Serum (FBS; Life Technologies, USA). 293T human embryonic kidney cells were maintained in Opti-MEMI (Life Technologies, Gaithersburg, MD, USA) containing 5% FBS for reassortant virus synthesis. After 48 h the transfected supernatants were collected and virus titers were determined by standard hemagglutination assays as described previously [21]. The sequences were confirmed using a specific set of universal primers as described previously [22]. Viruses were propagated in 10 day old specific pathogen free embroyonated chicken eggs at 37°C. The tissue culture infectious dose 50 (TCID50) of reassortant virus was then calculated by the Muench-Reed method (1938) [23].

For challenge experiments H7N7 reassortant virus was mouse adapted by three sequential lung to lung passages, as described previously [24]. Virus present in the lung passage 3 was propagated in the allantoic cavities of 10 days old chicken eggs for 48 h at 37°C to prepare a virus stock. The TCID50 and 50% mouse lethal dose (MLD50) were calculated as described by Reed and Munch et al., 1938 [23]. For the control vaccine group, the H7N7-RG virus was inactivated by BEI (binary ethylenimine) as described previously [25]. The complete loss of infectivity of the inactivated virus was determined by inoculation and titration of vaccine preparation in eggs. All experiments were performed in a biosafety level 3 (BSL-3) containment laboratory in compliance with CDC/NIH and WHO recommendations [26], [27] and were approved by the Agri Veterinary Authority (AVA) of Singapore.

### Preparation of recombinant baculovirus subunit vaccine

The recombinant baculovirus vector was generated as described previously [10]. The full length HA gene was amplified from H7N7 (A/NL/219/03) reassortant virus in a standard PCR reaction (94°C 20 s, 55°C 30 s and 72°C 2 min for 30 cycles) with the primer set: HA forward XbaI 5′TGC**TCTAGA**GCAGATGAACACTCAAATCCTG3′ and HA reverse Xh0I 5′CCG**CTCGAG**CGGTTATATACAAATAGTGCAC3′. The amplified HA gene was inserted into the shuttle vector pFASTBacHT A (Invitrogen, San Diego, CA, USA) for expression under the white spot syndrome virus (WSSV) immediate early (Ie1) promotor. This expression cassette was integrated into the baculovirus genome within DH10Bac^TM^ (Invitrogen, USA) through site specific transposition according to the protocol of the Bac-to-Bac system (Invitrogen). SF9II cells were maintained in SF900II serum free medium (Gibco BRL, USA) at 28°C for recombinant baculovirus synthesis. The recombinant bacmid was then transfected into SF9II cells and the supernatant containing recombinant baculovirus displayed H7N7-HA (Bac-HA) was harvested at 96 h post-infection. The supernatant was centrifuged at 500×g for 10 min and the virus titer was determined by a standard plaque assay with SF9 cells according to the baculovirus construction protocol (Invitrogen, No.10359). The vaccine was prepared based on a hemagglutination titer (HAI) of 2^8^. The Bac-HA was inactivated with binary ethylenimine (BEI) as described previously [28] for the use as vaccine control in this experiment.

### Immunofluorescence and Western blot assays to detect HA expression in insect cells

For Western blot analysis the recombinant baculovirus or Bac-wt infected cell supernatants were subjected to 12% SDS gel electrophoresis. The gel was transferred to a nitrocellulose membrane. For HA protein detection, polyclonal H7N7-HA mouse antibody was used as primary antibody (1∶1000 dilution) and rabbit anti-mouse IgG conjugated to HRP monoclonal antibody (Dakocytomation, Denmark) at a dilution of 1∶1000 was used as secondary antibody. The protein bands were visualized by the ECL chemiluminescence kit (Amersham, UK).

For immunofluorescence assay the Sf9 cells were cultured in six well plates and infected with Bac-HA at 0.5 MOI. Two days post infection the cells were fixed with 4% PFA for 20 min at room temperature then rinsed with PBS and blocked with 5% milk powder in PBS containing 0.05% Tween 20 (PBS-T) for 1 h at room temperature. Then the cells were incubated with primary antibody against H7N7-HA (8H9 monoclonal antibody, 1∶100 dilution) for 1 h at 37°C followed by three washes with PBS-T and two washes with PBS. The cells were subsequently incubated with the secondary antibody (FITC conjugated goat anti mouse IgG, 1∶50 dilution (DakoCytomation, Denmark)). Negative control cells were treated the same way. The fluorescence signal was detected with an inverted fluorescence microscope (Olympus, UK) and the images were captured by a digital imaging system (Nikon, USA).

### Mice immunization and challenge

Six to eight weeks old female BALB/c specific pathogen free mice were used in all experiments. Mice (25 mice per group/10 groups) were vaccinated by intranasal or subcutaneous route. Before immunization, preimmune serum and nasal wash samples were collected. The procedure for intranasal administration has been described previously [10]. Mice were immunized with 100 μL containing 2^8^ HA units of adjuvant free, purified live Bac-HA, inactivated Bac-HA, inactivated H7N7 reassortant virus (IV), or wild-type baculovirus (Bac-wt; 10^8^ PFU) and PBS control. For s.c. immunization, mice were immunized with live Bac-HA, inactivated Bac-HA or inactivated H7N7 influenza virus (IV) in the presence of Montanide ISA 201 VG adjuvant, or Bac-wt and PBS at the same concentrations as by i.n. route. A second dose of the same concentration of vaccine was given to all i.n. and s.c. immunized mice groups 4 weeks after the first immunization. Five mice from each experimental group were sacrificed 14 days after the first and second immunization and the sera and nasal washes were collected as described previously [29]. All samples were stored at −20°C before measuring antibody levels, microneutralization and hemagglutinin inhibition.

To assess the protective efficacy of the vaccines, immunized groups were challenged with mouse adapted H7N7 virus 3 weeks after the final immunization. 15 mice per group of each vaccination experiment were anesthetized by ketamine/xylazine and infected intranasally with mouse adapted H7N7 reassortant virus at 5 MLD50 per mouse, administered in a 100 μL dose. Mice were weighted daily and disease symptoms were analysed until all control mice died or until 16 days post challenge. Mice that lost more than 25% of their body weight were euthanized.

### Systemic and mucosal immune response

The serum IgG and mucosal IgA-specific antibody levels were measured against H7N7-HA antigen separately by indirect enzyme linked immunosorbent assay (ELISA). Each well in the ELISA plates was coated with 100 μL (1 mg/ml in PBS) purified recombinant H7N7-HA protein in 100 μL of coating buffer (0.1 mol/l carbonate/bicarbonate, pH 9.6) and incubated overnight at 4°C. Antigen-coated plates were washed with PBS containing 0.05% PBS-T and non-specific sites were blocked with 100 μL blocking buffer (PBS containing 5% skim milk) for 1 h at 37°C. Test sera and nasal washes were diluted at 1∶100 and 1∶50 respectively in PBS-T and 100 μL was added to triplicate wells and incubated for 1 h at 37°C followed by three washes with PBS-T. 100 μL of goat anti-mouse IgG (Sigma, USA) or IgA (Bethyl Lab) conjugated with horseradish peroxidise diluted 1∶1000 in PBS were added to the respective wells. The plates were incubated at 37°C for 1 h and washed three times with PBS-T. The colour reaction was developed by the addition of 100 μL of 3, 3′, 5, 5′-tetramethyl benzidine (Sigma, USA) and the reaction was stopped after 5–10 min with 25 μL of 1 M sulphuric acid. The absorbance was measured at 450 nm using an ELISA plate reader (Tecan, Sunriser, Switzerland). The mean absorbance value for triplicate wells was used to express serum antibody levels.

### Microneutralization assay and HAI

The serum neutralizing antibody titers were determined by microneutralization assay [10]. MDCK cells were seeded at 1×10^4^ cells/well in 96-well culture plates and cultured at 37°C for 24 h to form a monolayer. Serial two fold dilutions of heat inactivated (56°C for 45 min) 42nd day serum samples were mixed separately with 100 TCID50 of H7N7 reassortant virus and were incubated at room temperature for 1 h. The mixtures were added to the MDCK monolayers in triplicate wells. The neutralizing titers of mouse antisera that completely prevented cytopathic effect (CPE) at reciprocal dilutions were calculated. Functional serum antibody titers were also determined by hemagglutination inhibition assay [21]. Receptor destroying enzyme (RDE Denka Siken Co., Japan) treated sera were serially diluted (2-fold) in V-bottom 96-well plates. Approximately 4 HA units of viral antigen was incubated with the serum for 30 min at room temperature, followed by the addition of 1% chicken RBCs and incubation at room temperature for 40 min. The inhibition of hemagglutination at the highest serum dilution was considered the HI titer of the serum.

### ELISPOT assay

On day 8 post-challenge, three mice from each group were sacrificed and their spleens were harvested to count H7N7 specific IFN-γ and IL-4 secreting cells by ELISPOT assay [30]. Splenocytes were obtained by straining the spleens through a 70 μm cell strainer (BD Falcon, USA) and red blood cells were lysed in lysis buffer (0.15 M NH_4_Cl, 1 M K_2_CO_3_, and 0.01 M EDTA, pH 7.2) for 10 min at room temperature. Splenocytes were washed and resuspended in RPMI medium. The viability of cells was determined by trypan blue staining and live cells were counted with a hemocytometer. 2×10^5^ live cells per well were added to a 96-well multiscreen HTS immuno-plate (Millipore, USA) pre-coated with anti-mouse IL-4 or IFN-γ monoclonal antibody (Ebiosciences, USA). Cells were stimulated with 1 μL/well pytohemagglutinin (PHA; Invitrogen, USA) as a positive control or 5 μL/well of 10^4^ TCID50/ml of H7N7 influenza virus overnight in a 37°C incubator with 5% CO2. Unstimulated wells served as negative controls. All conditions were examined in triplicates. Secreted cytokines were detected by the addition of anti- IL-4 or IFN-γ secondary antibodies (100 μL/well) coupled to biotin and avidin-HRP according to the manufacturer's protocol (Ebiosciences, USA). Plates were developed by addition of 3-amino-9-ethyl-carbazole (AEC; BD Biosciences, USA) for 10 min. Spot development was stopped by washing with distilled water. Spot forming units (SFU) were counted in an immuno-spot analyzer (Cellular Technology Limited, USA).

### Quantification of influenza virus in lungs by real time RT-PCR

On day 8 post-challenge, three mice from each group were sacrificed and their lungs were harvested and homogenized in 1 mL PBS. RNA was extracted from the homogenate by TRIzol method following the manufacturer's protocol (Invitrogen, USA). The viral load of the lungs was quantified by real time RT-PCR method using Rotor GeneQ (Qiagen Inc., USA) and Quanti fast SYBR® Green master mix (Qiagen, USA). The cDNA conversion and amplification were performed in a single step with a primer pair targeting the matrix gene M2 of PR8 virus. A standard curve and cycle threshold values were obtained using serial dilutions of the matrix gene cloned into pGEMT vector. All data were calibrated relative to β-actin mRNA levels as an endogenous internal control. Each assay was carried out in triplicates and viral loads were quantified by the following equation: (6.02×1023 copies/mol) × (concentration of plasmid g/mL)/(MW g/mol)  =  copies/mL.

### Histopathological analysis

Lung samples for histological examination were harvested from each mice group (N = 3) on day 8 post challenge and fixed in 10% buffered formalin (pH 7.4), embedded in paraffin and sectioned at 4 µm. The sections were deparaffinized using Hist-choice (Amersco) and rehydrated in sequentially graduated ethanol baths. The slides were stained with hematoxylin and eosin and pathological evaluation was performed by light microscope (Olympus, UK). The images were captured by digital imaging system (Nikon, USA).

### Statistical analysis

All data were expressed as arithmetic mean ± standard deviation. The significance between the groups was calculated using unpaired two-tailed Student's t test and was expressed as * p<0.05.

## Results

### H7N7 reassortant virus synthesis and mouse adaptation

Reassorted virus H7N7 was successfully generated according to the plasmid based reverse genetics method as described previously [20]. The sequence of each gene segment of the reassortant virus was confirmed to be identical to the sequence of the corresponding gene in the parent virus. Reassorted virus was then grown in cell culture and eggs and TCID50 was measured (10^3.16^). A previous study has shown that H7N7/NL/219 parental virus strain can replicate and cause mortality in mice without prior adaptation. We assessed the replicative capability and virulence of reassorted H7N7 NL/219 virus strain in mice by i.n. inoculation with 50 μL of virus. All mice were observed for clinical signs of illness like ruffled fur, weight loss and hunching shoulders for a 16 day period. On day 7, moderate clinical symptoms with a 10% weight loss were observed but no mortality was recorded as all mice recovered from the infection. The virulence of the reassortant H7N7 RG virus was subsequently increased by serial lung to lung passages. At day 8 post inoculation, all mice lost up to 20% of their body weight and died at day 10. The lung supernatants were then propagated in the allantoic cavity of eggs, and the TCID50 was quantified. The viral titer of this lung-passaged H7N7 was 10^4.36^ TCID50/ml, representing a 1.2 log increase compared to the original reassortant virus titer. To calculate the lethal dosage of H7N7, different TCID50 dilutions were inoculated i.n. into mice, and the MLD50 was determined to be 10^2.6^/ml.

### Baculovirus-HA vaccine construction

**Figure 1:**
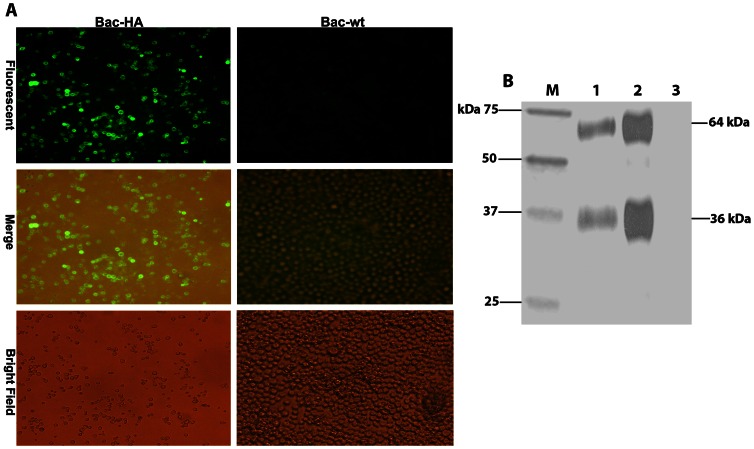
Confirmation and characterization of baculovirus surface displayed HA (Bac-HA) of H7N7 influenza virus by (A) immunofluorescence assay and (B) Western blot. (A) The Sf9 cells were infected with baculovirus expressed HA from H7N7 (Bac-HA) or wild type baculovirus (Bac-wt) at a MOI of 0.5. 48 h post infection the cells were fixed and analyzed by mAb specific to H7N7 influenza virus to determine the structural and antigenic confirmation of baculovirus expressed HA. (B) Western blot analysis of Bac-HA showing the cleavage of HA using anti-mouse HA polyclonal antibody. Lane M: broad range protein molecular weight marker; Lane 1: Bac-HA infected supernatant; Lane 2: H7N7 reassortant influenza virus, Lane 3: Bac-wt infected supernatant.

The recombinant baculovirus (Bac-HA) was constructed to express the H7N7-HA protein on the baculovirus surface as described in materials & methods. The expression of HA in Bac-HA infected Sf9-II cells was analysed by immunofluorescence assay in which fluorescence signal was detected only on the surface of the baculovirus expressed HA (Bac-HA) infected cells. In contrast, no fluorescence signal was observed in cells infected with Bac-wt or in uninfected cells (Fig. 1A). The molecular mass of HA was confirmed by Western blotting using an anti-mouse H7N7-HA polyclonal antibody, in which two bands with molecular masses of around 64 kDa and 37 kDa corresponding to HA0 and HA1 were detected. There were no bands detected in the Bac-wt infected cell supernatants (Fig. 1B). Further, we examined whether the baculovirus surface displayed HA was properly folded and exhibited hemagglutination activity. Based on the HA titer assay, Bac-HA infected supernatant showed a clear agglutination activity of up to 2^8^ HA units. As expected, no agglutination was observed in Bac-wt infected supernatant. Based on the above results, we confirmed that the HA proteins expressed in insect cells were translocated to the cell surface, were properly folded and exhibited hemagglutination activity.

### Systemic and immune response

**Figure 2:**
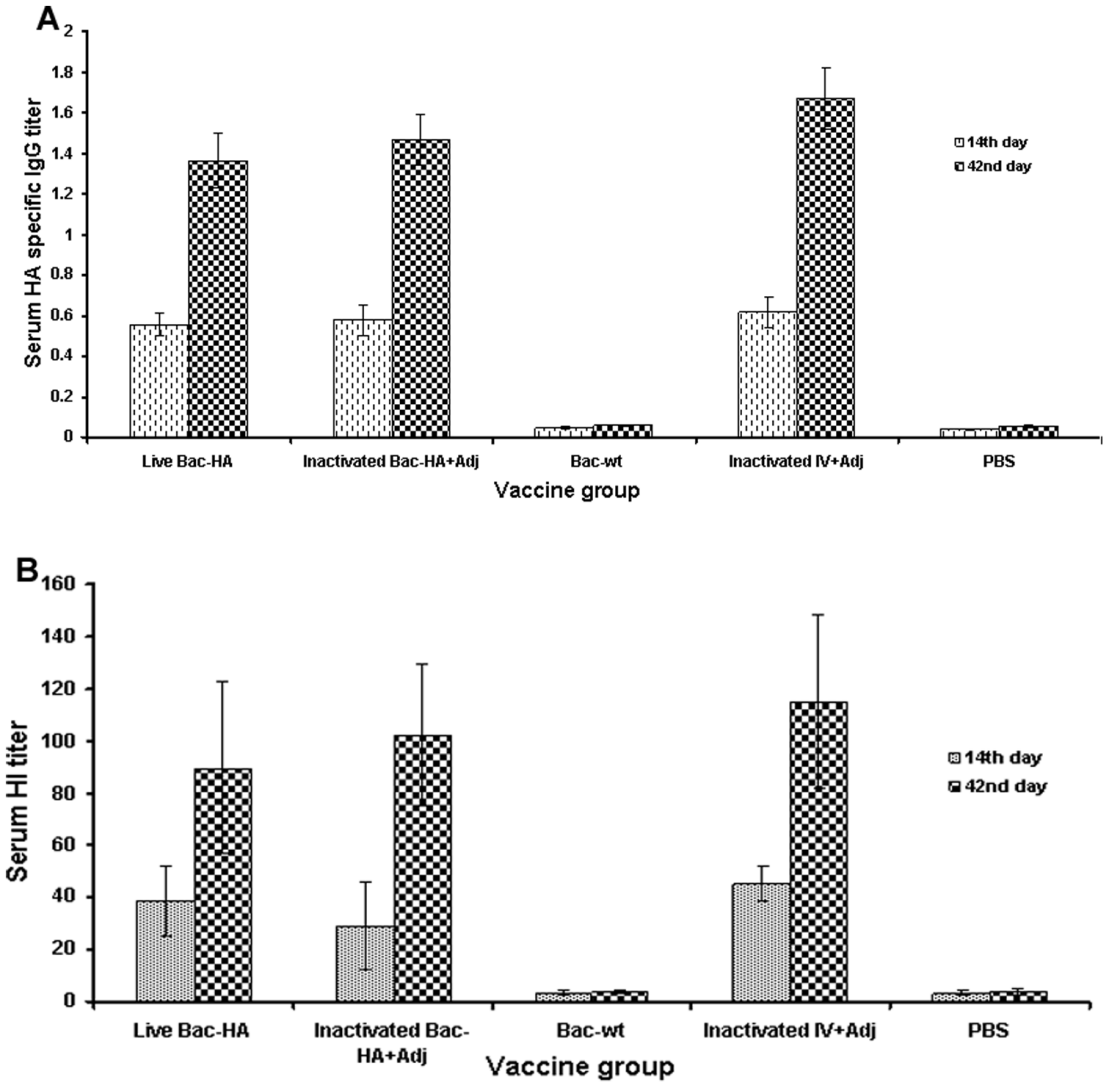
Measurement of systemic immune responses of mice sera obtained from subcutaneous immunizations on days 14 and 42. Groups of mice (n = 10) were immunized with live Bac-HA, inactivated Bac-HA+Adj, inactivated influenza virus (IV)+Adj, Bac-wt and PBS control on day 0 and 28. A constant dosage of 100 μL of 2^8^ HA titer of Bac-HA or inactivated IV suspended in PBS was administrated. The sera were used to determine the HA-specific IgG antibody titer by indirect ELISA (A) and the serum hemagglutination inhibition (HI) titer (B). Each point represents the arithmetic mean value (n = 5) ± SD.

**Figure 3:**
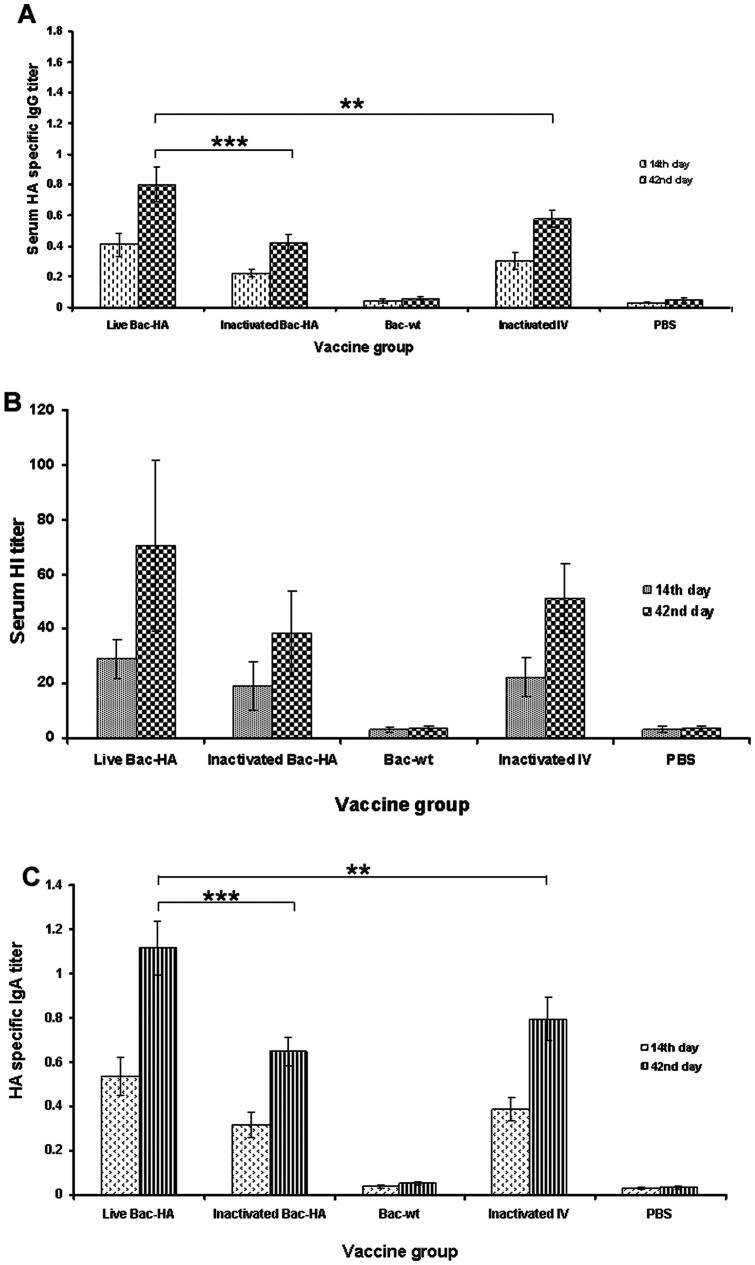
Measurement of systemic and mucosal immune responses of mice sera obtained from intranasal immunizations on days 14 and 42. Groups of mice (n = 10) were intranasally immunized with live Bac-HA, inactivated IV, Bac-wt, inactivated Bac-HA or PBS controls on day 0 and 28. A constant dosage of 100 μl of 2^8^ HA titer of Bac-HA or inactivated IV suspended in PBS was administrated. The sera were used to determine the HA-specific IgG antibody titer by indirect ELISA (A), the serum hemagglutination inhibition (HI) titer (B), and the HA-specific IgA titer by indirect ELISA (C). Each point represents the arithmetic mean value (n = 5) ± SD. **, P<0.001; ***, P<0.0001.

Systemic and mucosal immune responses were evaluated in mice sera and nasal washes obtained from s.c. or i.n. vaccinated mice groups on days 14 and 42. Vaccinated groups and sample collections were described in materials and methods. The Bac-HA vaccine efficacy was evaluated by measuring the induction of H7N7-HA specific serum IgG by indirect ELISA and the activity of serum antibodies by hemagglutination inhibition and microneutralization assays. Further, mucosal IgA antibody responses against HA protein were analyzed in i.n. immunized mice groups. The sera of mice immunized s.c. with adjuvanted inactivated IV exhibited a higher IgG titer compared to other s.c. or i.n. immunized mice groups. However, no significant difference was observed between mice immunized s.c. with live Bac-HA or adjuvanted inactivated Bac-HA (Fig. 2A). Even though IgG levels were lower in the i.n. than in the s.c. immunized groups, the live Bac-HA group had the highest IgG levels among the i.n. vaccinated groups and a significant difference was observed between live Bac-HA and inactivated IV or inactivated Bac-HA at day 42 (Fig. 3A, P<0.002). The serum IgG levels of PBS control mice or Bac-wt mice were negligible regardless of the immunization route.

The functionality of these serum antibodies was further tested in a hemagglutination inhibition (HI) assay. Sera of mice immunized s.c. with adjuvanted inactivated IV showed slightly enhanced HI titer compared to live Bac-HA immunized mice sera, but no significant difference between the groups was observed (Fig. 2B). Also, no significant difference was observed between the HI titers of serum from mice immunized s.c. with inactivated IV, live Bac-HA, or adjuvanted inactivated Bac-HA. While sera from the mice immunized intranasally with live Bac-HA showed a higher HI titer compared to the other i.n. immunized mice groups on day 42 this increase was not significant (Fig. 3B). Further, we observed that live Bac-HA induced a slightly higher HI titer in mice immunized s.c. compared to i.n. albeit not significantly. In both vaccination experiments, a very low HI titer was recorded for Bac-wt and PBS control sera.

Further, the HA specific mucosal IgA in the nasal washes of i.n. immunized mice was measured by indirect ELISA. The IgA level was significantly elevated in samples collected from live Bac-HA groups at day 42 compared to both inactivated IV or inactivated Bac-HA immunized mice (Fig. 3C, P<0.0001). The IgA titers of the Bac-wt and PBS control groups were negligible. The ability of the serum antibodies to neutralize live influenza virus was tested in a microneutralization assay. The neutralizing antibody titers of sera from s.c. and i.n. immunized groups were measured at day 42 against 100 TCID50 of mouse adapted H7N7 reassortant virus (Table 1). The sera obtained from the i.n. vaccinated live Bac-HA group showed a higher neutralizing titer against H7N7 virus compared to the other vaccinated groups. This indicates that mucosal IgA significantly contributes to efficient virus neutralization ability in the immunized mice.

**Table 1:** Virus neutralizing antibody titers of subcutaneous or intranasal immunized mice sera against 100 TCID50 of H7N7 reassortant influenza virus.

Immunization route/groups	Serum neutralization antibody titer
** A) Subcutaneous immunization**	
1) Live Bac-HA	40
2) Inactivated IV+Adj	40
3) Inactivated Bac-HA +Adj	40
4) Bac-wt	0
5) PBS	0
** B) Intranasal immunization**	
1) Live Bac-HA	160
2) Inactivated IV	40
3) Inactivated Bac-HA	40
4) Bac-wt	0
5) PBS	0

### Cellular immune response

**Figure 4:**
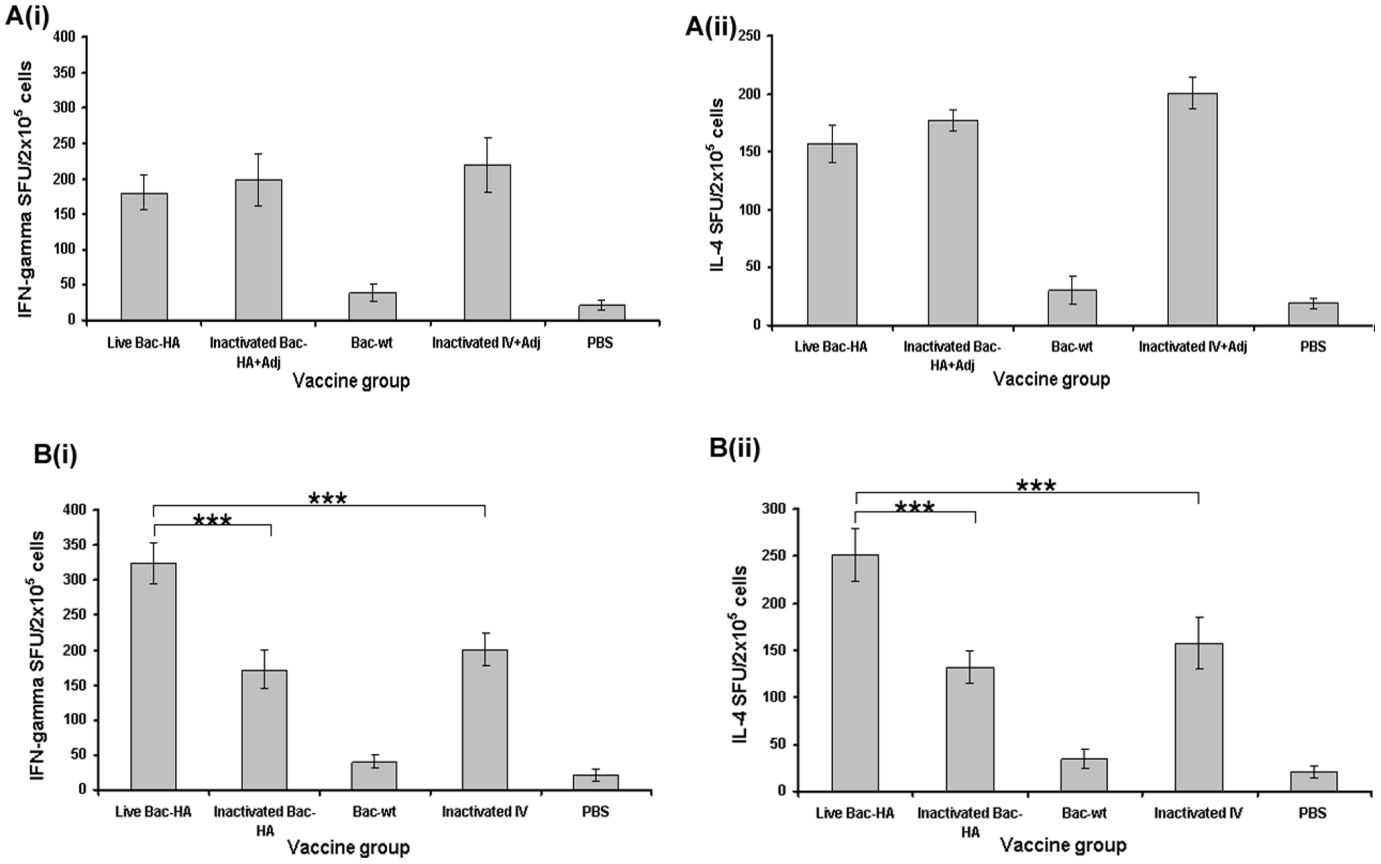
ELISPOT results of IFN-γ and IL-4 responses in subcutaneously A (i), A (ii) and intranasally immunized mice B (i), B (ii). Spleen cells were isolated on 8 dpi from the various mice groups (n = 3/group) and were pooled for the determination of IFN-γ and IL-4 responses. The splenocytes were stimulated overnight with live H7N7 reassortant influenza virus. Each point represents the arithmetic mean value (n = 3) ± SD. The statistical significance of the difference between groups was calculated by student's t test. ***, P<0.0001. SFU – spot-forming units.

The cellular immune response elicited by the vaccine candidates was determined by the activity of Th1 (IFN-γ) and Th2 cytokine (IL-4) secreting cells in spleens using ELISPOT assay. As shown in figure 4B (i), mice vaccinated by i.n. live Bac-HA had a significantly higher IFN-γ response (300–350 spots/2×10^5^ cells) than other i.n. and s.c. vaccinated groups (Fig. 4A(i)). Similarly, IL-4-secretion (250–275 spots/2×10^5^ cells) was significantly higher in the intranasally live Bac-HA vaccinated mice group (Fig. 4B (ii)) than in the other i.n. or s.c. vaccinated groups (Fig. 4B (i)). Bac-wt vaccinated mice splenocytes had T cell responses (IFN-γ and IL-4) similar to the negative control PBS mice. The higher level of IFN-γ and IL-4 secretion of splenocytes is an indicator of the activity of functional cytotoxic T cells responsible for clearing the virus-infected cells. These results suggest that intranasal vaccination with live Bac-HA not only triggered a mucosal and system antibody response but also effectively stimulated Th1 and Th2 immune responses.

### Mice challenge study

**Figure 5:**
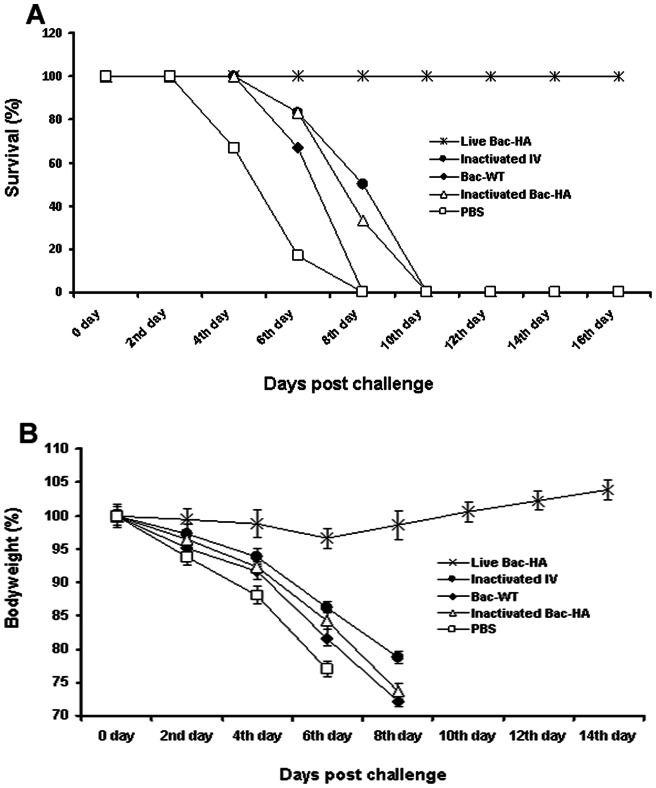
Protection of mice from lethal H7N7 influenza virus challenge. **Mice (n = 6/groups) were immunized intranasally on day 0 and 28.** All groups were challenged intranasally with 5 MLD50 of mouse-adapted H7N7 virus on day 49. Mice were monitored for survival for 16 days and the results were expressed in percent survival (A). Weight loss of the mice groups was also monitored throughout the 16 day observation period and the results were expressed in percent body weight compared to the beginning of the trial (B).

**Figure 6:**
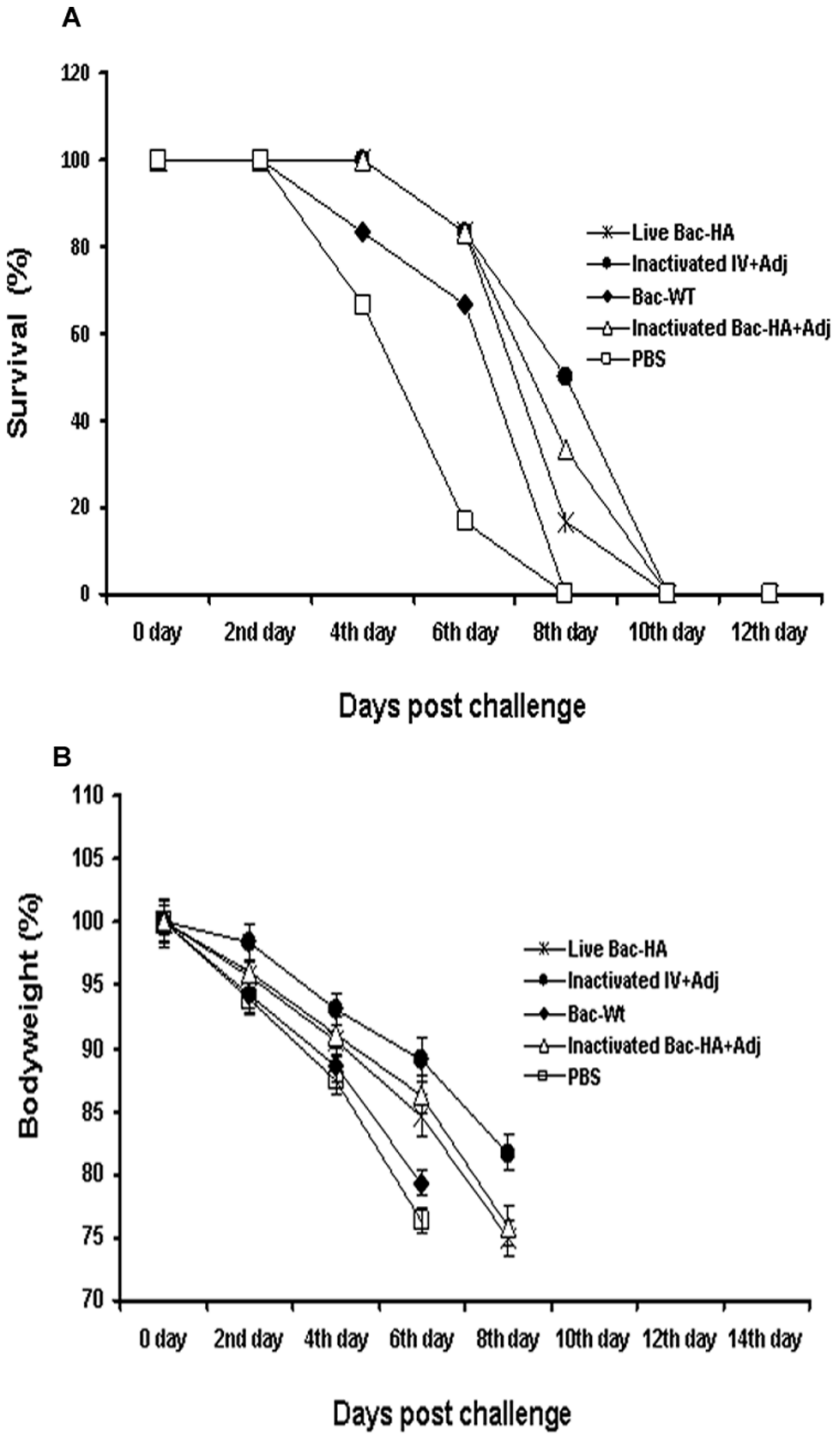
Protection of mice from lethal H7N7 influenza virus challenge. Mice (n = 6/groups) were immunized subcutaneously on day 0 and 28. All groups were challenged intranasally with 5 MLD50 of mouse-adapted H7N7 virus on day 49. Mice were monitored for survival for 16 vdays and the results were expressed in percent survival (A). Weight loss of the mice groups was also monitored throughout the 16 day observation period and the results were expressed in percent body weight compared to the beginning of the trial (B).

**Figure 7:**
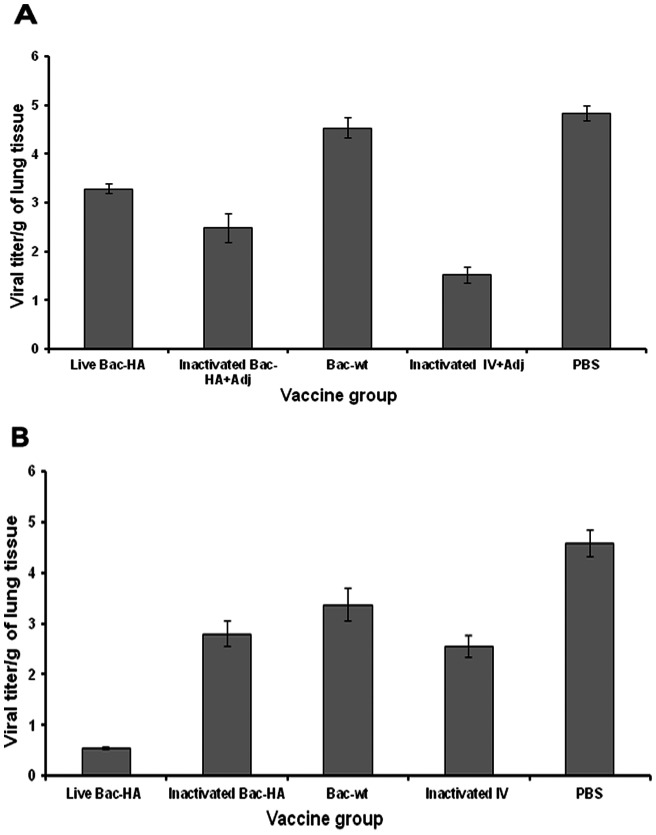
H7N7 influenza viral titers in lungs at 8 day post challenge following subcutaneous (A) or intranasal (B) immunization. Viral titers were measured by quantitative real time RT-PCR using influenza virus M2 gene primers. The results were expressed in terms of log TCID50/g ± SD.

**Figure 8:**
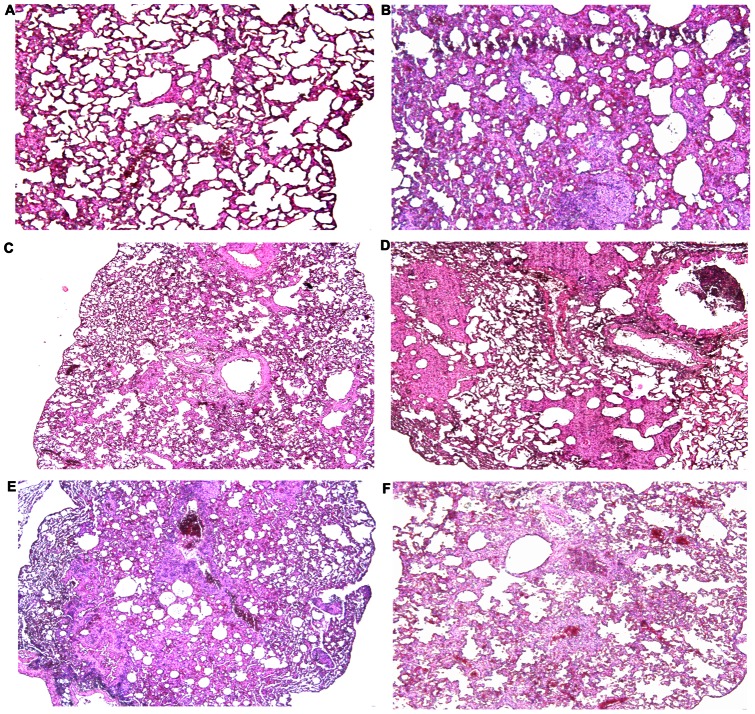
Histopathological changes in lung sections of mice challenged with a lethal dose of H7N7 virus at day 8 after subcutaneous or intranasal immunizations. (A) Intranasal immunization with live Bac-HA. The mice had minimal or no lesions in the examined lung section. (B)–(F) Subcutaneous immunizations with live Bac-HA (B); inactivated IV+Adj (C); inactivated Bac-HA+Adj (D); live Bac-wt (E); PBS (F). (B)–(D) Mice present with moderate alveolar collapse throughout the lung tissue. (E)–(F) Both groups display severe alveolar collapse throughout the lungs tissue.

Four weeks after the final immunization all mouse groups were challenged with 50 μL containing 5 MLD50 of mouse adapted H7N7 reassortant influenza virus. Even though the earlier results showed that s.c. immunization with live Bac-HA induced a higher level of IgG response compared to i.n. immunization, 100% protection (Fig. 5A) was achieved only in mice immunized intranasally with live Bac-HA throughout the 16 day observation period with a negligible weight loss (Fig. 5B). All the other vaccine constructs, including inactivated adjuvanted IV administered by s.c. route, failed to protect the mice (Fig. 6A). These animals showed obvious clinical symptoms such as ruffled fur and hunching shoulders at day 4 post challenge. Also, a significant weight loss of up to 25% to 35% was observed before they were sacrificed at day 10 (Fig. 6B). Three mice per group were sacrificed at day 8 post challenge to determine the H7N7 influenza virus titer in the mice lung tissues by quantitative real time RT-PCR. Results showed that the H7N7 influenza virus titer was significantly reduced in the i.n. live Bac-HA immunized group compared to the other vaccinated groups (Fig. 7A and 7B). Additionally, the histopathological changes in the lungs of immunized mice and control mice were examined 8 days post challenge. The lung tissue of negative control mice showed local inflammation in the lungs and invagination of the pulmonary pleura, alveolar collapse and cell debris in the bronchiolar lumen, as well as lung deformation with enlargement of the alveolar duct. The same pathological changes were observed in the lungs of mice immunized s.c. by live Bac-HA or by adjuvanted inactivated IV or inactivated Bac-HA (Fig. 8B, C, and D) but the damage was less severe compare to the Bac-wt and PBS negative controls (Fig. 8E and 8F). In contrast, the lungs of i.n. live Bac-HA immunized mice showed minimal bronchitis and an absence of lesions (Fig. 8A).

## Discussion

H7N7 (NL/219/03) virus has come into focus as a major pandemic concern because of its ability to transmit directly from domestic poultry to humans and from human to human [2]. The FC H7N7 possesses a similar affinity towards the respiratory tract as H5N1 and its antiviral inhibition property is similar to pandemic 1918 H1N1 and 2009 H1N1 [4] influenza viruses, which may facilitate this virus as the next pandemic causative agent. The generation of an effective vaccine against H7N7 virus is highly desirable. In this present study we developed a recombinant HA vaccine (Bac-HA) against H7N7 virus by using the baculovirus expression vector system. Also, we generated a reassortant influenza virus H7N7 (NL/219/2003) which was adapted in mice to evaluate the protective efficacy of the recombinant baculovirus surface displayed H7N7-HA (Bac-HA) vaccine in a challenge study. Previous reports showed that the wild type H7N7 (NL/219/03) replicates efficiently in mice and causes mortality without prior adaptation because the virus possesses Lys at PB2-627 which conveys special advantages to replicate efficiently in the upper and lower respiratory tracts of mammals similar to H5N1 viruses [3]. However reassortant H7N7 virus required three lung passages in mouse before causing mortality. Sequencing of mice adapted RG virus did not uncover any sequence differences in the HA, NA and PB2 gene between parental H7N7 or PR8 viral genes (data not shown). However, after adaptation the TCID50 of adapted virus showed a 1.2 log increase compared to the original reassortant H7N7 virus. A further detailed study is needed to address this issue.

The baculovirus system has already been shown to display several immunogenic proteins in their functional forms which have been used successfully as vaccines against several diseases [17], [18], [19]. Accordingly, we analysed whether the HA protein of H7N7 virus, expressed on the baculovirus envelope, exhibits the same functional activity as HA of H7N7 virus. Based on the authentic cleavage of expressed HA into HA0 and HA1 and on its hemagglutination activity, we confirmed that the surface-expressed HA retained the native confirmation of HA of wild type H7N7 virus.

Baculoviruses have strong adjuvant activity to promote humoral and cellular immune responses against coadministered antigens [31]. Based on the ELISA data, we confirmed that intranasally administered recombinant live Bac-HA induced a higher systemic IgG and mucosal IgA response against specific H7N7-HA compared to other i.n. vaccinated groups. However, the HA specific serum IgG level was higher in all s.c. immunized groups at 42 days post immunization. Similarly, we observed that the HI titer was higher in the sera from the mice immunized s.c compared to mice vaccinated i.n., and higher in i.n. live Bac-HA compared to the other i.n. groups, however these differences were not statistically significant. These results reconfirmed that subcutaneous (parenteral delivery) immunization can elicit higher antibody responses in systemic sites than intranasal vaccination. Further, we examined the mucosal IgA levels elicited by the mice intranasally immunized with live Bac-HA. The intranasal live Bac-HA immunized group had a significantly higher level of HA specific mucosal IgA compare to the other i.n. immunized groups. This could be either due to the ability of the live recombinant baculovirus to efficiently transduce the HA gene into the host tissue or due to the adjuvant properties of the unmethylated CpG motif present in the baculoviral DNA [14]. In contrast, inactivated Bac-HA vaccine induced low levels of humoral and cellular immune responses, most likely because inactivated vaccine was unable to transduce cells due to the degradation of its genome caused by inactivation with BEI [32], [33]. Hence inactivation strongly abolished the adjuvant properties of baculovirus and resulted in a poor immune response against the coadministred antigen [34]. Further, we observed that the neutralizing activity of mice sera induced by s.c. vaccination with live Bac-HA or adjuvanted inactivated vaccines was low which is in agreement with a previous study reporting that Dutch H7 viruses are poor inducers of neutralizing antibodies [6]. On the other hand, the neutralizing activity of i.n. immunized live Bac-HA sera against the H7N7 reassortant influenza virus was significantly enhanced at day 42 post immunization compared with the neutralizing activity of the other i.n. or s.c. immunized mice groups. The reason for the enhanced neutralization activity of sera from the intranasally immunized mice could be due to the induction of both HA specific mucosal IgA and serum IgG levels or due to live baculovirus acting as a strong adjuvant that can activate the DCs and macrophages, which are abundant in nasal mucosal tissues, against coadministered antigens [14]. In addition, several previous reports also indicated that the neutralizing efficacy of IgA antibodies may be greater than that of IgG [35], [36].

Next we investigated HA specific IFN-γ and IL-4 levels in the splenocytes of immunized mice. Results showed that i.n. live Bac-HA immunized mice splenocytes secreted significantly higher amounts of IFN-γ and IL-4 compared to s.c. live Bac-HA immunized mice splenocytes. Previous reports also reported that intranasal immunization induces higher amounts of IL-4 and IFN-γ than subcutaneous immunization [37], [38]. The reason for the enhanced cytokine level in splenocytes of the i.n. immunized mice could be due to the recall response in splenocytes upon nasal challenge with influenza virus. Also, Kiayno et al [39] reported that the presence of a unique immune component of the nasal mucosa (nasopharynx-associated lymphoid follicles) can confer induction of antigen-specific Th lymphocytes, CTLs, and IgA B-cell responses. In contrast, stimulated splenocytes from inactivated Bac-HA mice secreted lower levels of IFN-γ and IL-4 compared to live Bac-HA immunized mice splenocytes. It has been reported that live baculovirus exhibited antiviral activity in mammalian cells by inducing IFN-γ and other proinflammatory cytokines [40]. This robust cellular immune response suggested that live Bac-HA acted as both subunit and live viral vaccine and induced both Th1 and Th2 biased immune responses in mice which are associated with the Toll-like receptor 9 (TLR9)-dependent pathway [31]. Hence, the balance between Th1 and Th2 cytokines is required for the protection against H7N7 pathogen.

The protective efficacy of the vaccine candidates was determined in a mouse challenge study. Results showed that mice vaccinated intranasally with live Bac-HA showed 100% protection against lethal H7N7 influenza virus, whereas no protection was observed in mice vaccinated with any of the other vaccine candidates. Even though the results showed that mice immunized s.c. with live Bac-HA produced higher IgG and similar HI titer level as the i.n. immunized mice, the neutralization titer of the later was elevated 4-fold over the other vaccine candidates (Table 1). Hence, neutralization and protection correlated perfectly. The lack of additional immune responses like mucosal IgA could be one of the reasons for the failure of s.c. immunization in protecting against lethal influenza H7N7 viral challenge. Renegar et al [41] findings suggested that IgA is more important than IgG in the protection of the upper respiratory tract (RT) infection, whereas IgG is more important than IgA in the protection of the lungs in the lower RT infection. HA is the viral surface glycoprotein responsible for attachment and fusion; avian influenza viruses preferentially bind to α 2-3-linked sialic acids (SA), whereas the HA of human influenza viruses preferentially binds to α 2-6-linked SA which is predominantly expressed on the apical side of the upper respiratory epithelia. Kyoko Shinya et al [42] and Debby van Riel et al [43] reported that seasonal and pandemic human influenza viruses preferentially recognize SA α 2-6-Gal receptor and infected epithelial cells lining the bronchi and alveolar cells which is in the upper region of the respiratory tract [44]–[46]. Viral growth in the upper respiratory tract may provide a platform for the adaptation of influenza viruses to humans and for efficient person-to-person virus transmission [47]. The surface glycoprotein genes of HA and NA of our mouse adapted H7N7 reassortant virus, used in the vaccine challenge, were taken from a fatal case of human infection. Previous evidence showed that it can transmit from human to human indicating efficient growth in the upper respiratory tract [2]. Belser et al [48] also found that avian HP H7N7 viruses from the Netherlands in 2003 maintained the classic avian-binding preferences for α 2-3-linked sialic acids, like highly pathogenic H5N1 viruses. Masato Hatta et al [47] suggested that viruses possessing Lys at PB2-627 have special advantages to replicate efficiently in the upper and lower respiratory tracts of mammals again similar to H5N1 viruses. Munster et al [3] also supported that the fatal case H7N7 NL/03 virus replicated well in the upper and lower respiratory tracts of mice and ferrets because it has Lys at pB2-627 position. Based on our results and these previous reports we conclude that both mucosal IgA and serum IgG are important to protect the mice against H7N7 NL/03 influenza virus infection.

Further, we investigated the viral titer in lung tissues of challenged mice. Results showed that the viral copy number is significantly lower in i.n. live Bac-HA immunized mice lung tissue compared to all other immunized groups. The protection could be due to the successful induction of mucosal IgA, serum IgG, IFN-γ and IL-4 immune responses which prevented the attachment or replication of the virus in the upper and lower respiratory tract of the challenged mice. Abe et al [14] reported that the lungs of mice inoculated with baculovirus exhibited a marked infiltration of macrophages, which presumably can inhibit the growth of influenza virus in the lung tissues. Next, we examined the lung tissues of the challenged mice by histopathology at 8 days. The i.n. live Bac-HA immunized mice lungs had minimal or no lesions in the lung tissue, whereas the lungs of the other infected mice groups showed severe infection with cell debris in the bronchiolar lumen, followed by enlargement of the alveolar ducts and severe alveolar collapse in the whole lung as previously demonstrated by Fukushi et al [49]. The histology result therefore support the finding that live Bac-HA induced a high level of antiviral responses and protected the mice against mouse adapted HP H7N7 virus.

In summary, we observed that intranasal vaccination with live Bac-HA induced both humoral and cellular immune responses in systemic and mucosal sites and protected the mice from HP H7N7 reassortant influenza virus infection. As adjuvanted, inactivated virus immunized s.c. did not protect against a lethal H7N7 challenge, the only other possible vaccine would be an intranasally administered live attenuated influenza virus. However, live attenuated viruses carry the risks of reversion or recombination with circulating seasonal flu. Producing live Bac-HA is also a much safer option as it does not require either sophisticated bio containment infrastructure or purification processes for mass production during pandemic or prepandemic situations. From a HP H7N7 protection standpoint, intranasal vaccination with live Bac-HA subunit vaccine would be a promising alternative to the live and inactivated vaccine approach.
